# DNA methylation in the inflammatory genes after neurosurgery and diagnostic ability of post-operative delirium

**DOI:** 10.1038/s41398-021-01752-6

**Published:** 2021-12-09

**Authors:** Takehiko Yamanashi, Takaaki Nagao, Nadia E. Wahba, Pedro S. Marra, Kaitlyn J. Crutchley, Alissa A. Meyer, Ally J. Andreasen, Mandy M. Hellman, Sydney S. Jellison, Christopher G. Hughes, Pratik P. Pandharipande, Matthew A. Howard, III, Hiroto Kawasaki, Masaaki Iwata, Marco M. Hefti, Gen Shinozaki

**Affiliations:** 1 Stanford University School of Medicine, Department of Psychiatry and Behavioral Sciences, Palo Alto, CA USA; 2 University of Iowa Carver College of Medicine, Department of Psychiatry, Iowa City, IA USA; 3 Tottori University Faculty of Medicine, Department of Neuropsychiatry, Yonago-shi, Tottori Japan; 4 University of Iowa Carver College of Medicine, Department of Neurosurgery, Iowa City, IA USA; 5 Toho University School of Medicine Faculty of Medicine, Department of Neurosurgery (Sakura), Sakura-shi, Chiba Japan; 6 Vanderbilt University Medical Center, Department of anesthesiology, Nashville, TN USA; 7 University of Iowa Carver College of Medicine, Department of Pathology, Iowa City, IA USA

**Keywords:** Epigenetics and behaviour, Molecular neuroscience

## Abstract

The pathophysiological mechanisms of postoperative delirium (POD) are still not clear, and no reliable biomarker is available to differentiate those with and without POD. Pre- and post-surgery blood from epilepsy subjects undergoing neurosurgery were collected. DNA methylation (DNAm) levels of the *TNF* gene, *IL1B* gene, and *IL6* gene by the Illumina EPIC array method, and DNAm levels of the *TNF* gene by pyrosequencing, were analyzed. Blood from 37 subjects were analyzed by the EPIC array method, and blood from 27 subjects were analyzed by pyrosequencing. Several CpGs in the *TNF* gene in preoperative blood showed a negative correlation between their DNAm and age both in the POD group and in the non-POD group. However, these negative correlations were observed only in the POD group after neurosurgery. Neurosurgery significantly altered DNAm levels at 17 out of 24 CpG sites on the *TNF* gene, 8 out of 14 CpG sites on the *IL1B* gene, and 4 out of 14 CpG sites on the *IL6* gene. Furthermore, it was found that the Inflammatory Methylation Index (IMI), which was based on the post-surgery DNAm levels at the selected five CpG sites, can be a potential detection tool for delirium with moderate accuracy; area under the curve (AUC) value was 0.84. The moderate accuracy of this IMI was replicated using another cohort from our previous study, in which the AUC was 0.79. Our findings provide further evidence of the potential role of epigenetics and inflammation in the pathophysiology of delirium.

## Introduction

Delirium is an acute and devastating illness that can be caused by various medical and surgical conditions or drugs. Postoperative delirium (POD) occurs in up to 50–70% of high-risk patient groups [1] and is associated with prolonged hospital stay [1–3], higher rates of mortality [1–4], and institutionalization after discharge [5, 6]. Although prevention, early detection, and effective treatment for delirium are important, their effectiveness is limited. Part of the reason is due to a lack of a clear understanding of the pathophysiological mechanisms of delirium, and no reliable biomarker is available to differentiate those with and without delirium. Thus, there is an urgent need to identify biomarkers of delirium risk and to better elucidate the pathophysiology of delirium.

Accumulating evidence suggests that systemic and neuroinflammation play a key role in the development of delirium [7–10]. Many human studies have investigated inflammatory cytokines in delirium subjects [11, 12]. Although a meta-analysis showed increased levels of IL-6 in blood from POD patients, the evidence for other inflammatory markers is still unclear [11, 12].

We have previously reported several epigenetic studies that investigated DNA methylation (DNAm) levels of delirium subjects [13–16]. For example, it was shown that DNAm levels in several CpG sites on the *TNF* gene were negatively correlated with age only in delirium subjects [16]. We also identified a genome-wide significant CpG at the *LDLRAD4* gene [14]. Furthermore, an enrichment analysis demonstrated significant differences related to the immune/inflammatory response pathways between patients with delirium and non-delirium controls [14]. These evidences suggest that DNAm levels, especially at inflammatory-related genes, can be a potential biomarker for delirium. However, no pre- and post-delirium sample to compare was available in the previous studies. Thus, no information was obtainable if those DNAm changes were present before the onset of delirium (as an indicator of their baseline vulnerable state), or if those DNAm alterations occurred after delirium or etiological disease conditions such as infection or surgery (as a marker of disease state). Also, the data mentioned above was based on delirium subjects from diverse medical/surgical conditions and thus prone to have many uncontrollable confounders.

Here, we hypothesized that surgical invasion alters DNAm levels in inflammatory-related genes, which causes systemic inflammation followed by POD, and that altered inflammation-related DNAm levels can be used as a biomarker to detect POD. In order to test this hypothesis and overcome the challenges from our previous studies, in the present study, we collected blood from pre- and post-neurosurgery patients and examined levels of DNAm in inflammatory-related genes, the *TNF, IL1B, and IL6*, at each time point pre- and post-surgery. We investigated correlations between age and DNAm levels. We also compared DNAm levels of POD patients and ones of non-POD subjects to find a biomarker for POD. Furthermore, we investigated how neurosurgery altered DNAm levels to explore epigenetic mechanisms of POD. Finally, by using significant DNAm differences at several CpG sites, we construct a score to differentiate POD versus non-POD, and tested against an independent sample from our previous cohort to test the performance of such a scoring method.

## Patients and methods

### Participants

Thirty-nine subjects with medically intractable epilepsy undergoing neurosurgery were recruited between April 2015 and September 2020 at the University of Iowa Hospitals and Clinics. Eighteen of them were subjects who were recruited in our previous studies [17, 18]. Participants formed a consecutive series. This study was approved by the University of Iowa Human Subjects Office Institutional Review Board. We obtained written informed consent from all participants.

### Clinical assessment and case definition

Briefly, we obtained demographic information from electronic medical records (EMRs) and patient interviews. We reviewed data on each subject using EMRs to capture if there was evidence of delirium after the surgery. POD-positive cases were identified based on if they tested positive on the Confusion Assessment Method for the Intensive Care Unit (CAM-ICU) [19] or had clinical documentation of altered mental status or confusion consistent with delirium from the EMR. The CAM-ICU was administered by nursing staff. A board-certified consultation-liaison psychiatrist (G.S.), who was blinded to methylation status, reviewed each patient record for final determination of case categorization in question.

### Sample collection and processing

Preoperative blood samples were collected in the operating room at the beginning of surgery preparation when intravenous lines were placed following our standard protocol. Thus, this was certainly before the start of the surgical procedure. Postoperative blood samples were again collected in the operating room after the procedure but before awakening from anesthesia. We collected whole blood samples in EDTA tubes. Blood cells were stored at −80 °C until DNA extraction.

### DNA extraction and bisulfite conversion

As previously described [14–16, 18], DNA was isolated from blood with the MasterPureTM DNA extraction kit (Epicenter, MCD85201) following the respective protocols. DNA quality was assessed with NanoDrop spectrometry and quantity was assessed with the Qubit™ dsDNA Broad Range Assay Kit (ThermoFisher Scientific, Q32850). DNA was stored at −80 °C until bisulfite conversion. DNA was bisulfite-converted using the EZ DNA Methylation™ Kit (Zymo Research, D5002). Bisulfite-converted DNA was stored at −80 °C until epigenetics analysis. DNA was used for EPIC analysis first, and the remainder of the DNA was used for pyrosequencing. Pyrosequencing was not conducted when enough DNA was not available after EPIC analysis.

### A genome-wide analysis using Illumina EPIC array

DNAm of bisulfite-converted blood DNA samples were analyzed with the Infinium HumanMethylationEPIC BeadChip™ Kit (WG-317-1002, Illumina) as previously described [14–16, 18]. The samples were placed on EPIC array chips randomly to avoid potential confounding batch effects. Thus, there were no distortion of sample placement across those chips. DNAm data of 24 CpG sites on the *TNF* gene, 14 CpG sites on the *IL1B* gene, and 14 CpG sites on the *IL6* gene were extracted and analyzed.

### Nested polymerase chain reaction (PCR) and pyrosequencing

Thirty-two CpG sites in the *TNF* gene were targeted in our pyrosequencing analyses as previously described [16]. Nested PCRs were conducted using bisulfite-converted DNA samples as previously described [16]. After nested PCRs, pyrosequencing was performed using PyroMark Q96 (Qiagen, #972804) and PyroMark Q24 Pyrosequencer (Qiagen, #9001514) to detect levels of DNA methylation.

### Statistical analysis

Statistical analyses were performed using R [20]. The student’s *t*-test was performed to compare DNAm levels between the POD group and the non-POD group. Multiple regression analysis was also performed to compare POD and non-POD; results of the EPIC analysis were corrected for age, sex, and cell distribution, and results of pyrosequencing were corrected for age and sex. Paired *t*-test was performed to compare DNAm levels pre-surgery and post-surgery. Pearson’s correlation analysis was performed to calculate correlations between age and DNAm levels of each CpG sites. After correction for multiple testing, *P* values <0.05 (EPIC; *P* < 0.05/52 = 0.00096, pyrosequencing; *P* < 0.05/32 = 0.00156) were considered statistically significant.

The receiver operating characteristic (ROC) curve with area under the curve (AUC) were used to analyze the relationship between DNAm level from post-surgery blood and POD. Five CpG sites from each gene were chosen based on the post-neurosurgery results and scores were given based on their best cut-off scores. A total score named “Inflammatory Methylation Index (IMI)” (ranging from 0 to 5) was obtained for each subject, and AUC based on the ROC curve was calculated to evaluate the relationship between IMI and POD. IMI using the same five CpG sites was also obtained from our previous delirium study cohort [14, 16], and a ROC curve was made to test the performance of this scoring method for the detection of delirium. Furthermore, IMIs using other five CpG sites were obtained from DNAm level from pre-surgery blood and amount of change in DNAm level after surgery, and ROC curves were made to test the performance of this scoring method for prediction of POD. Details of IMI calculation are described in the supplementary method.

## Results

### Study subject demographics

Totally, 39 subjects were enrolled in this study; 37 subjects (10 POD subjects and 27 non-POD subjects) were included in the EPIC array analysis, and 27 subjects (7 POD subjects and 20 non-POD subjects) were included in pyrosequencing analysis. Demographic information is shown in Supplementary Table 1. Mean age was higher in the POD group than in the non-POD group. Sex, race, alcohol usage, tobacco usage, American Society of Anesthesiologists (ASA) physical status, body mass index (BMI), anesthesia time, procedure time, or amount of bleeding was not different between the POD group and non-POD group (Supplementary Table 1). There were no significant differences in CD8 T cells, CD4 T cells, natural killer cells, B cells, and monocytes between the POD group and the non-POD group (Supplementary Table 2).

### DNA methylation before surgery

#### TNF gene

Data from EPIC analysis demonstrated that there was no difference of DNAm level in the *TNF* gene before surgery between the POD group and the non-POD group (Supplementary Table 3). Negative correlations between age and DNAm level were observed in several CpG sites on the *TNF* gene with statistical significance (Table 1). These negative correlations were observed both in the POD group and in the non-POD group (Table 1). Pyrosequencing analysis demonstrated the same tendency. There was no difference in DNA methylation level before surgery between the POD group and the non-POD group (Supplementary Table 3). Negative correlations between age and DNAm were observed in several CpG sites on the *TNF* gene with nominal significances (Table 1). These negative correlations were observed both in the POD group and in a non-POD group (Table 1).

**Table 1:** Correlations between age and pre-surgery DNAm in the *TNF* gene.

		EPIC		EPIC		EPIC		pyrosequencing		pyrosequencing		pyrosequencing	
		all		POD		non-POD		all		POD		non-POD	
		*N* = 37		*N* = 10		*N* = 27		*N* = 27		*N* = 7		*N* = 20	
cg	location	r	*p*	r	*p*	r	*p*	r	*p*	r	*p*	r	*p*
	chr6: 31542460							−0.43	0.03	−0.57	0.18	−0.29	0.22
cg08639424	chr6: 31542556	−0.18	0.28	−0.21	0.56	−0.17	0.39						
cg19978379	chr6: 31542671	0.28	0.10	0.01	0.98	0.40	0.04						
cg24452282	chr6: 31542741	0.22	0.18	0.14	0.70	0.27	0.17						
	chr6: 31543170							−0.47	0.01	−0.62	0.14	−0.42	0.06
	chr6: 31543176							−0.46	0.02	−0.70	0.08	−0.41	0.07
	chr6: 31543178							−0.54	0.004	−0.62	0.14	−0.39	0.09
	chr6: 31543193							−0.51	0.006	−0.56	0.19	−0.49	0.028
cg21370522	chr6: 31543219	−0.17	0.32	−0.03	0.93	−0.29	0.15	−0.49	0.009	−0.57	0.18	−0.47	0.035
cg19648923	chr6: 31543266	−0.40	0.01	−0.32	0.37	−0.44	0.02	−0.38	0.051	−0.54	0.22	−0.31	0.18
cg01569083	chr6: 31543290	−0.39	0.02	−0.29	0.41	−0.47	0.01	−0.53	0.005	−0.64	0.12	−0.46	0.040
cg03037030	chr6: 31543300	−0.47	0.004	−0.43	0.21	−0.47	0.01	−0.40	0.04	−0.53	0.23	−0.37	0.11
	chr6: 31543430							−0.48	0.01	−0.41	0.36	−0.41	0.08
	chr6: 31543487							−0.45	0.02	−0.33	0.46	−0.42	0.07
cg12681001	chr6: 31543541	−0.52	*0.0009	−0.42	0.22	−0.58	0.0014	−0.38	0.049	−0.13	0.78	−0.37	0.10
cg21222743	chr6: 31543546	−0.60	*<0.0001	−0.66	0.04	−0.57	0.0018	−0.42	0.03	−0.20	0.67	−0.42	0.07
cg10717214	chr6: 31543558	−0.58	*0.0002	−0.70	0.02	−0.54	0.0038	−0.02	0.94	0.11	0.83	−0.07	0.79
cg04425624	chr6: 31543566	−0.50	0.0016	−0.62	0.06	−0.50	0.0081	−0.24	0.26	−0.22	0.68	−0.30	0.24
cg21467614	chr6: 31543638	−0.64	*<0.0001	−0.70	0.03	−0.66	*0.0002						
cg08553327	chr6: 31543647	−0.57	*0.0003	−0.63	0.05	−0.56	0.0023						
cg26729380	chr6: 31543655	−0.62	*<0.0001	−0.71	0.02	−0.59	0.0013						
cg10650821	chr6: 31543686	−0.46	0.0039	−0.37	0.29	−0.52	0.0056						
	chr6: 31544695							−0.03	0.89	0.11	0.82	0.01	0.98
	chr6: 31544752							−0.12	0.56	−0.04	0.92	−0.24	0.31
	chr6: 31544800							−0.05	0.80	−0.20	0.67	−0.18	0.46
	chr6: 31544822							−0.05	0.82	0.13	0.78	−0.24	0.31
	chr6: 31544848							−0.24	0.22	−0.76	0.047	−0.34	0.14
cg01360627	chr6: 31544931	−0.09	0.60	−0.11	0.75	−0.08	0.70	−0.11	0.60	−0.19	0.68	−0.29	0.22
cg23384708	chr6: 31544934	−0.01	0.96	0.06	0.87	−0.27	0.17	−0.01	0.96	−0.03	0.95	−0.24	0.31
cg20477259	chr6: 31544960	0.01	0.97	0.15	0.69	−0.13	0.53	0.03	0.89	0.01	0.98	−0.03	0.89
	chr6: 31545253							−0.02	0.92	0.52	0.23	−0.20	0.39
	chr6: 31545258							−0.11	0.58	0.27	0.56	−0.36	0.11
cg15989608	chr6: 31545322	−0.21	0.20	−0.31	0.38	−0.25	0.21	−0.11	0.57	0.25	0.59	−0.40	0.08
	chr6: 31545267							−0.09	0.66	0.13	0.80	−0.38	0.11
cg26736341	chr6: 31545343	−0.18	0.28	−0.24	0.51	−0.26	0.20	0.28	0.17	0.51	0.30	0.28	0.24
	chr6: 31545432							0.10	0.63	0.23	0.62	−0.12	0.62
cg04472685	chr6: 31545474	−0.11	0.53	−0.15	0.68	−0.14	0.49	−0.32	0.15	0.18	0.77	−0.46	0.06
cg19124225	chr6: 31545836	−0.08	0.63	0.02	0.96	0.01	0.97						
cg02137984	chr6: 31545899	−0.34	0.04	−0.40	0.25	−0.21	0.29						
cg06825478	chr6: 31546068	0.10	0.54	−0.17	0.63	0.14	0.48	0.21	0.29	0.44	0.32	0.15	0.52
	chr6: 31546086							0.00	0.99	0.22	0.64	−0.10	0.67

underline nominal significance (*P* < 0.05).

*Significant after correction for multiple testing level (EPIC; *P* < 0.05/52 = 0.00096, pyrosequencing; *P* < 0.05/32 = 0.00156).

#### IL1B and IL6

There was no difference in DNA methylation level before surgery between the POD group and the non-POD group (Supplementary Table 4). No CpG site showed a correlation between age and DNAm level with statistical significance (Supplementary Table 5).

### DNA methylation after surgery

#### TNF gene

The EPIC analysis demonstrated that there was no significant difference in DNAm level after surgery between the POD group and the non-POD group (Supplementary Table 6). Moderate negative correlations between age and DNAm level, observed in pre-surgery samples, were observed in several CpG sites (from chr6: 31543546 to chr6: 31543655) on the *TNF* gene in the POD group, but not in the non-POD group (Table 2).

**Table 2:** Correlations between age and post-surgery DNAm in the *TNF* gene.

		EPIC		EPIC		EPIC		pyrosequencing		pyrosequencing		pyrosequencing	
		all		POD		non-POD		all		POD		non-POD	
		*N* = 37		*N* = 10		*N* = 27		*N* = 27		*N* = 7		*N* = 20	
cg	location	r	*p*	r	*p*	r	*p*	r	*p*	r	*p*	r	*p*
	chr6: 31542460							0.09	0.67	0.01	0.98	0.13	0.60
cg08639424	chr6: 31542556	−0.03	0.84	−0.50	0.14	−0.08	0.69						
cg19978379	chr6: 31542671	0.17	0.33	−0.14	0.70	0.19	0.34						
cg24452282	chr6: 31542741	0.19	0.25	0.00	0.99	0.17	0.40						
	chr6: 31543170							−0.33	0.10	−0.44	0.32	−0.54	0.01
	chr6: 31543176							–0.43	0.02	-0.33	0.47	−0.53	0.02
	chr6: 31543178							−0.21	0.29	−0.08	0.87	−0.22	0.34
	chr6: 31543193							−0.49	0.01	−0.61	0.14	−0.45	0.046
cg21370522	chr6: 31543219	0.26	0.13	0.23	0.51	0.28	0.16	−0.34	0.08	−0.44	0.32	−0.46	0.04
cg19648923	chr6: 31543266	0.00	0.98	0.00	0.99	0.02	0.91	−0.26	0.18	−0.39	0.39	−0.33	0.16
cg01569083	chr6: 31543290	0.06	0.71	−0.09	0.80	0.18	0.36	−0.47	0.01	−0.30	0.51	−0.55	0.01
cg03037030	chr6: 31543300	−0.03	0.84	−0.18	0.61	0.07	0.73	−0.29	0.14	0.03	0.96	−0.40	0.08
	chr6: 31543430							−0.44	0.02	−0.60	0.16	−0.41	0.07
	chr6: 31543487							−0.38	0.05	−0.48	0.28	−0.33	0.16
cg12681001	chr6: 31543541	−0.02	0.89	0.09	0.81	0.02	0.93	−0.12	0.54	−0.18	0.69	−0.17	0.47
cg21222743	chr6: 31543546	−0.36	0.03	−0.84	0.003	−0.22	0.28	−0.33	0.09	−0.18	0.69	−0.40	0.08
cg10717214	chr6: 31543558	−0.29	0.08	−0.57	0.09	−0.22	0.27	0.37	0.09	0.24	0.70	0.34	0.18
cg04425624	chr6: 31543566	−0.16	0.35	−0.34	0.33	−0.04	0.85	0.10	0.65	−0.85	0.07	0.30	0.25
cg21467614	chr6: 31543638	−0.33	0.04	−0.56	0.09	−0.22	0.27						
cg08553327	chr6: 31543647	−0.24	0.15	−0.37	0.30	−0.13	0.53						
cg26729380	chr6: 31543655	−0.37	0.03	−0.51	0.13	−0.26	0.20						
cg10650821	chr6: 31543686	−0.16	0.35	0.03	0.93	−0.24	0.22						
	chr6: 31544695							0.02	0.93	−0.25	0.59	0.21	0.37
	chr6: 31544752							0.06	0.77	0.65	0.11	−0.06	0.79
	chr6: 31544800							0.27	0.18	0.28	0.55	0.05	0.82
	chr6: 31544822							0.18	0.36	0.36	0.43	0.15	0.53
	chr6: 31544848							−0.07	0.72	−0.11	0.82	−0.33	0.16
cg01360627	chr6: 31544931	−0.05	0.79	−0.21	0.57	−0.09	0.67	0.16	0.44	0.04	0.92	0.05	0.85
cg23384708	chr6: 31544934	0.16	0.35	0.31	0.38	0.06	0.77	0.29	0.14	0.64	0.12	0.13	0.58
cg20477259	chr6: 31544960	0.07	0.69	0.08	0.82	0.01	0.96	0.25	0.21	0.36	0.43	0.13	0.57
	chr6: 31545253							0.09	0.67	−0.29	0.53	0.22	0.34
	chr6: 31545258							0.01	0.95	0.33	0.48	−0.12	0.62
	chr6: 31545267							0.16	0.43	0.17	0.72	0.20	0.39
cg15989608	chr6: 31545322	0.04	0.80	−0.26	0.47	0.05	0.81	0.10	0.60	0.52	0.23	0.16	0.51
cg26736341	chr6: 31545343	0.05	0.78	−0.36	0.31	0.10	0.61	0.06	0.77	0.53	0.22	−0.18	0.44
	chr6: 31545432							0.07	0.73	−0.28	0.54	0.05	0.83
cg04472685	chr6: 31545474	0.00	0.99	0.20	0.57	−0.08	0.68	−0.05	0.84	−0.44	0.39	0.07	0.80
cg19124225	chr6: 31545836	0.07	0.70	0.51	0.13	−0.20	0.32						
cg02137984	chr6: 31545899	−0.03	0.87	−0.24	0.50	−0.02	0.91						
cg06825478	chr6: 31546068	−0.15	0.37	−0.12	0.75	−0.22	0.27	0.31	0.12	0.38	0.40	0.30	0.20
	chr6: 31546086							0.18	0.38	0.24	0.61	0.17	0.48

underline nominal significance (*P* < 0.05).

Pyrosequencing analysis did not demonstrate any DNA methylation differences between the POD group and the non-POD group (Supplementary Table 6). In the pyrosequencing analysis, negative correlations between age and DNAm were observed in several CpG sites on the *TNF* gene both in the POD group and in the non-POD group (Table 2).

#### IL1B and IL6

There was no difference in DNA methylation level after surgery between the POD group and the non-POD group (Supplementary Table 7). No CpG site showed a correlation between age and DNA methylation with statistical significance (Supplementary Table 8).

### Change of DNA methylation by neurosurgery

Among 24 CpG sites on the *TNF* gene measured by EPIC analysis, 11 CpG sites showed a significant increase of DNA methylation and six CpG sites showed a significant decrease of DNA methylation after neurosurgery in all subjects (Table 3). The same patterns of DNAm change were shown in pyrosequencing. Eight CpG sites showed a significant increase of DNAm level and one CpG site showed a significant decrease of DNA methylation after neurosurgery (Table 3). DNAm in most of CpG sites upstream from chr6: 31542741 and downstream from chr6: 31544695 increased after neurosurgery. Otherwise, DNAm in CpG sites from chr6: 31543219 to chr6: 31543686 decreased after neurosurgery. However, the degree of DNAm changes by the neurosurgery between the POD group and non-POD group were not different with statistical significance either in EPIC analysis or in pyrosequencing (Supplementary Table 9).

**Table 3:** Changes of DNAm in TNF gene after neurosurgery.

		EPIC				EPIC				EPIC				pyrosequencing				pyrosequencing				pyrosequencing			
		*N* = 37				*N* = 10				*N* = 27				*N* = 27				*N* = 7				*N* = 20			
		all				POD				non-POD				all				POD				non-POD			
		pre	post		paired*-t*	pre	post		paired-*t*	pre	post		paired*-t*	pre	post		paired*-t*	pre	post		paired*-t*	pre	post		paired*-t*
cg	location	mean	mean	diff	*p* value	mean	mean	diff	*p* value	mean	mean	diff	*p* value	mean	mean	diff	*p* value	mean	mean	diff	*p* value	mean	mean	diff	*p* value
	chr6: 31542460													74.0	78.1	4.1	*0.0004	72.7	78.1	5.4	0.10	74.5	78.1	3.6	0.00158
cg08639424	chr6: 31542556	77.9	86.2	8.3	*<0.0001	77.7	88.0	10.3	0.001	78.0	85.5	7.5	*<0.0001												
cg19978379	chr6: 31542671	71.8	81.0	9.2	*<0.0001	73.0	81.9	8.9	0.001	71.3	80.6	9.3	*<0.0001												
cg24452282	chr6: 31542741	73.5	84.5	11.1	*<0.0001	73.8	86.9	13.1	*<0.0001	73.3	83.6	10.3	*<0.0001												
	chr6: 31543170													7.0	5.7	−1.4	0.02	6.5	6.4	−0.1	0.94	7.2	5.4	−1.8	0.007
	chr6: 31543176													8.9	7.5	−1.3	0.03	8.0	7.5	−0.5	0.57	9.2	7.6	−1.6	0.04
	chr6: 31543178													3.3	2.6	−0.7	0.15	2.0	2.3	0.3	0.84	3.8	2.7	−1.1	0.03
	chr6: 31543193													5.2	4.0	−1.3	0.01	4.6	3.5	−1.1	0.32	5.5	4.1	−1.3	0.02
cg21370522	chr6: 31543219	13.2	14.1	0.9	0.34	13.6	14.4	0.8	0.64	13.0	13.9	1.0	0.41	4.2	3.5	−0.8	0.02	3.8	3.6	−0.2	0.75	4.4	3.4	−0.9	0.008
cg19648923	chr6: 31543266	10.6	8.1	−2.4	*0.0002	10.3	7.8	−2.5	0.005	10.6	8.2	−2.4	0.004	2.9	2.3	−0.6	0.02	2.7	2.4	−0.3	0.63	3.0	2.3	−0.8	0.02
cg01569083	chr6: 31543290	18.9	17.4	−1.5	0.14	19.0	16.8	−2.2	0.12	18.8	17.7	−1.2	0.36	3.3	2.6	−0.7	0.02	2.9	2.5	−0.4	0.55	3.5	2.7	−0.8	0.02
cg03037030	chr6: 31543300	6.3	4.9	−1.4	0.008	5.6	4.5	−1.1	0.06	6.6	5.0	−1.6	0.03	9.0	7.8	−1.2	0.02	8.5	7.6	−0.9	0.44	9.1	7.8	−1.3	0.02
	chr6: 31543430													1.9	2.0	0.0	0.97	1.5	1.9	0.4	0.42	2.1	2.0	−0.1	0.55
	chr6: 31543487													6.3	5.3	−1.0	0.046	5.3	4.9	−0.4	0.60	6.6	5.4	−1.2	0.06
cg12681001	chr6: 31543541	9.0	7.3	−1.8	0.002	8.8	6.7	−2.0	0.04	9.1	7.5	−1.7	0.02	10.4	9.1	−1.3	0.052	9.1	9.5	0.4	0.67	10.9	9.0	−1.9	0.02
cg21222743	chr6: 31543546	6.6	4.0	−2.6	*<0.0001	5.8	3.4	−2.4	0.003	6.9	4.2	−2.7	*0.0002	8.8	7.3	−1.5	0.01	7.7	7.1	−0.6	0.57	9.2	7.4	−1.8	0.01
cg10717214	chr6: 31543558	9.3	6.4	−2.9	*<0.0001	8.3	6.0	−2.2	0.002	9.7	6.5	−3.1	*0.0002	33.5	32.7	−0.8	0.47	33.6	35.0	1.4	0.48	33.5	32.0	−1.5	0.34
cg04425624	chr6: 31543566	17.4	13.1	−4.3	*0.0003	17.2	12.0	−5.2	0.01	17.5	13.5	−3.9	0.006	21.2	20.8	−0.4	0.86	20.8	21.6	0.8	0.57	21.3	20.5	−0.8	0.62
cg21467614	chr6: 31543638	17.7	14.7	−3.0	*<0.0001	17.1	14.1	−3.0	*0.0005	17.9	15.0	−2.9	0.003												
cg08553327	chr6: 31543647	16.5	13.1	−3.4	0.001	15.4	11.6	−3.8	0.003	16.9	13.7	−3.2	0.02												
cg26729380	chr6: 31543655	11.5	7.9	−3.7	*<0.0001	10.6	6.4	−4.2	0.005	11.9	8.4	−3.5	0.001												
cg10650821	chr6: 31543686	11.6	10.1	−1.5	0.007	11.4	10.0	−1.4	0.10	11.7	10.2	−1.5	0.03												
	chr6: 31544695													65.4	72.4	7.1	0.007	63.2	69.4	6.2	0.36	66.1	73.5	7.4	0.008
	chr6: 31544752													77.2	85.9	8.8	*<0.0001	78.2	86.2	8.0	0.10	76.8	85.9	9.0	*0.0004
	chr6: 31544800													22.7	24.4	1.7	0.01	24.0	26.3	2.3	0.25	22.3	23.8	1.5	0.02
	chr6: 31544822													56.6	62.2	5.6	*0.0005	58.1	62.5	4.4	0.13	56.0	62.1	6.1	0.002
	chr6: 31544848													13.3	13.3	0.0	0.95	14.6	14.0	−0.6	0.51	12.8	13.0	0.2	0.56
cg01360627	chr6: 31544931	70.0	78.6	8.7	*<0.0001	69.8	80.2	10.3	*<0.0001	70.0	78.1	8.0	*<0.0001	25.9	27.3	1.4	0.03	27.4	28.9	1.5	0.26	25.3	26.7	1.4	0.09
cg23384708	chr6: 31544934	64.1	72.2	8.1	*<0.0001	67.4	74.0	6.7	0.004	63.0	71.6	8.6	*<0.0001	74.0	82.5	8.5	*0.0001	77.3	83.9	6.6	0.18	72.8	82.0	9.2	*0.0004
cg20477259	chr6: 31544960	61.6	71.8	10.2	*<0.0001	62.5	73.4	10.9	*<0.0001	61.3	71.3	10.0	*<0.0001	67.9	74.7	6.8	*0.0004	68.9	76.4	7.5	0.09	67.5	74.1	6.6	0.003
	chr6: 31545253													88.1	91.9	3.8	0.003	87.9	92.3	4.4	0.17	88.2	91.8	3.6	0.010
	chr6: 31545258													87.7	91.6	3.8	0.009	88.7	92.2	3.5	0.25	87.4	91.3	3.9	0.02
	chr6: 31545267													82.0	87.8	5.8	*0.0005	84.1	87.8	3.7	0.33	81.3	87.8	6.5	*0.0005
cg15989608	chr6: 31545322	71.3	79.6	8.4	*<0.0001	72.2	80.8	8.7	0.001	70.9	79.1	8.2	*<0.0001	90.4	93.6	3.3	0.01	92.1	91.9	−0.2	0.83	89.8	94.2	4.4	*0.0006
cg26736341	chr6: 31545343	70.0	79.4	9.4	*<0.0001	71.4	80.6	9.3	*0.0008	69.5	79.0	9.4	*<0.0001	78.9	81.0	2.0	0.04	78.0	82.4	4.4	0.28	79.2	80.4	1.3	0.10
	chr6: 31545432													87.0	89.7	2.7	0.17	89.9	91.4	1.5	0.44	85.9	89.1	3.2	0.23
cg04472685	chr6: 31545474	81.4	87.5	6.1	*<0.0001	82.0	87.9	6.0	0.002	81.2	87.4	6.2	*<0.0001	84.0	86.8	2.8	0.008	83.4	87.1	3.6	0.31	84.2	86.7	2.5	0.02
cg19124225	chr6: 31545836	95.6	96.2	0.6	0.15	94.8	96.5	1.7	0.06	95.9	96.1	0.2	0.67												
cg02137984	chr6: 31545899	94.7	96.6	1.8	*0.0002	93.7	96.8	3.1	0.006	95.1	96.5	1.4	0.01												
cg06825478	chr6: 31546068	90.2	94.3	4.1	*<0.0001	91.0	94.7	3.6	0.01	89.9	94.1	4.2	0.0009	91.8	94.3	2.5	*<0.0001	91.8	94.5	2.6	0.02	91.8	94.2	2.4	*0.0014
	chr6: 31546086													85.1	89.3	4.2	*<0.0001	85.2	89.4	4.3	0.09	85.1	89.3	4.2	*0.0004

diff = post - pre.

underline nominal significance (*P* < 0.05).

*Significant after correction for multiple testing level (EPIC; *P* < 0.05/52 = 0.00096, pyrosequencing; *P* < 0.05/32 = 0.00156).

DNAm at eight out of 14 CpG sites on the *IL1B* gene and four out of 14 CpG sites on the *IL6* gene changed, most of them decreased, significantly after neurosurgery (Table 4). However, the degree of DNAm changes by the neurosurgery between the POD group and the non-POD group were not different with statistical significance (Supplementary Table 10).

**Table 4:** Changes of DNAm in the *IL1B* and *IL6* gene after neurosurgery.

	all				POD				non-POD			
	*N* = 37				*N* = 10				*N* = 27			
	pre	post	post-pre	paired*-t*	pre	post	post-pre	paired-*t*	pre	post	post-pre	paired*-t*
cg	mean	mean	diff	*p* value	mean	mean	diff	*p* value	mean	mean	diff	*p* value
*IL1B*												
cg01290568	26.4	16.1	−10.3	*<0.0001	26.2	12.7	−13.4	*<0.0001	26.5	17.3	−9.1	*0.0001
cg02596281	84.3	82.6	−1.7	0.01	84.4	81.8	−2.6	0.15	84.2	82.9	−1.3	0.05
cg07250315	37.7	33.2	−4.5	*<0.0001	39.9	33.0	−6.9	*0.0004	36.9	33.3	−3.6	0.004
cg07935264	9.0	6.5	−2.6	*0.0001	8.8	5.5	−3.2	0.02	9.2	6.8	−2.4	0.002
cg10486274	93.7	93.1	−0.6	0.09	93.9	93.1	−0.7	0.40	93.6	93.1	−0.5	0.15
cg14117934	86.3	85.5	−0.8	0.37	86.7	85.8	−0.9	0.41	86.1	85.4	−0.7	0.53
cg15218327	92.1	88.6	−3.5	*<0.0001	92.4	89.6	−2.8	0.01	92.0	88.2	−3.7	*<0.0001
cg15836722	26.3	20.1	−6.2	*<0.0001	27.3	17.1	−10.1	*0.0001	26.0	21.2	−4.7	0.003
cg18635064	94.6	94.3	−0.3	0.36	93.2	92.4	−0.8	0.26	95.1	95.0	−0.1	0.76
cg18773937	7.2	4.8	−2.3	*0.0001	6.8	4.2	−2.5	0.03	7.3	5.1	−2.3	0.002
cg19890119	15.6	8.8	−6.8	*<0.0001	15.9	6.7	−9.2	*<0.0001	15.5	9.6	−5.9	*0.0004
cg20157753	5.4	4.8	−0.6	0.06	5.9	4.2	−1.7	0.02	5.2	5.0	−0.2	0.56
cg20983042	87.1	87.0	0.0	0.95	87.5	86.8	−0.6	0.69	86.9	87.1	0.2	0.82
cg23149881	14.8	8.4	−6.3	*<0.0001	14.1	6.8	−7.4	0.003	15.0	9.1	−6.0	*0.0001
*IL6*												
cg00087425	6.4	6.6	0.2	0.62	6.3	6.2	−0.1	0.84	6.5	6.8	0.3	0.54
cg00175482	89.0	86.8	−2.2	0.002	88.6	85.7	−2.9	0.047	89.1	87.3	−1.9	0.02
cg01770232	7.1	7.6	0.5	0.09	7.7	7.7	0.1	0.90	6.9	7.5	0.6	0.04
cg02335517	93.4	92.7	−0.7	0.02	93.3	93.0	−0.3	0.42	93.4	92.5	−0.9	0.03
cg03601896	3.8	3.0	−0.8	0.02	4.0	2.7	−1.3	0.03	3.7	3.1	−0.6	0.14
cg05265849	20.2	17.6	−2.5	*0.0001	21.4	16.9	−4.5	*0.0005	19.7	17.9	−1.8	0.02
cg07998387	69.5	70.2	0.6	0.28	69.8	68.6	−1.3	0.31	69.4	70.7	1.4	0.04
cg10140158	87.7	87.9	0.2	0.70	86.8	87.0	0.2	0.79	88.0	88.2	0.2	0.77
cg13104385	39.9	39.1	−0.8	0.15	41.1	38.4	−2.6	0.02	39.5	39.4	−0.1	0.84
cg15703690	9.6	5.8	−3.8	*<0.0001	10.0	4.6	−5.4	0.001	9.5	6.2	−3.2	*0.0003
cg17067544	78.7	83.3	4.6	*<0.0001	79.3	85.0	5.7	0.01	78.5	82.7	4.2	*<0.0001
cg20509117	62.3	61.9	−0.4	0.68	62.2	62.5	0.2	0.90	62.3	61.6	−0.7	0.59
cg21785978	5.4	5.1	−0.3	0.44	5.2	4.4	−0.8	0.27	5.5	5.3	−0.1	0.78
cg23731304	37.6	34.5	−3.1	*0.0002	38.7	33.5	−5.2	0.005	37.2	34.9	−2.3	0.01

underline nominal significance (*P* < 0.05).

*Significant after correction for multiple testing level (EPIC; *P* < 0.05/52 = 0.00096).

### Inflammatory methylation index (IMI) and ROC curve

**Fig. 1:**
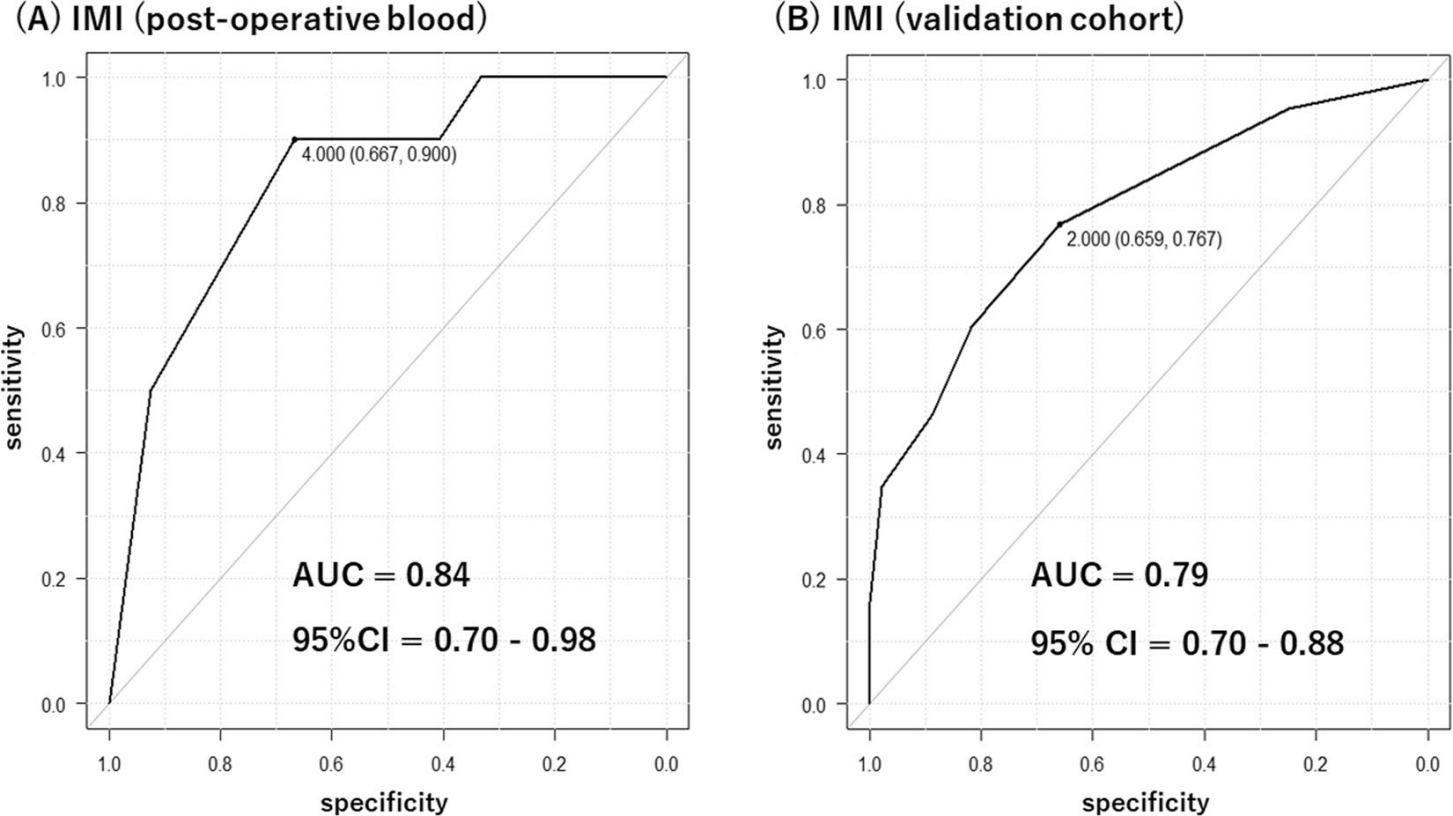
ROC curves based on IMI. (**A**) ROC curve based on IMI in the current dataset. (**B**) ROC curve based on IMI in validation dataset from our previous study.

We found that levels of DNAm at multiple CpG sites converged to a certain narrow range after surgery (Supplementary Fig. 1). Then, we calculated IMI (ranging from 0 to 5) based on the postoperative DNAm levels at the selected five CpG sites: one CpG site from the *TNF* gene (cg08639424), two CpG sites from the *IL1B* gene (cg15836722 and cg23149881), and two CpG sites from the *IL6* gene (cg15703690 and cg17067544). The AUC based on IMI was 0.84 (95% CI: 0.70–0.98) (Fig. 1A). We also calculated the IMI from the same five CpG sites using our previous delirium study cohort, including 87 subjects (43 delirium subjects and 44 control subjects) [14, 16], and tested the performance of the scoring using the ROC curve. The AUC based on the IMI was 0.79 (95% CI: 0.70–0.88) (Fig. 1B). Furthermore, the same approach was used to calculate the IMI from the preoperative DNAm (pre-IMI) and the amount of change in DNAm level after surgery (diff-IMI). Five different CpG sites were selected to optimize the ROC (Supplementary method and Supplementary Fig. 1). The AUC based on the pre-IMI was 0.92 (95% CI: 0.82–1.00) (Supplementary Fig. 2A), and the AUC based on the diff-IMI was 0.87 (95% CI: 0.73–1.00) (Supplementary Fig. 2B). IMI distributions are shown in Supplementary Fig. 3.

## Discussion

In this study, we investigated DNAm levels in inflammation-related genes using blood samples from patients who had neurosurgery. We examined correlations between age and DNAm level, compared peri-operative DNAm levels and postoperative ones, and compared DNAm levels in POD subjects with ones in non-POD subjects. We also constructed IMI aiming to differentiate POD from non-POD, validated with an independent cohort from our previous delirium study.

We have reported the relationship between delirium and DNAm [13–16]. The data from the Grady Trauma Project cohort demonstrated negative correlations between age and DNAm in several CpG sites on the *TNF* gene, but not in CpG sites on other inflammatory-related genes [13]. We also recently reported that delirium patients showed negative correlations between age and DNAm level in several CpG sites on the *TNF* gene [16]. In this current study, we demonstrated that DNAm levels in several CpG sites only on the *TNF* gene correlated with age using blood samples from all preoperative patients, consistent with our previous study [13]. Furthermore, negative correlations between age and DNAm levels on the *TNF* gene were observed after neurosurgery only in the POD group in EPIC analysis. This result is consistent with our previous study demonstrating negative correlations between age and DNAm level in several CpG sites on the *TNF* gene only in delirium subjects [16]. On the other hand, pyrosequencing showed negative correlations between age and DNAm levels after neurosurgery in both the POD group and the non-POD group. The discrepancy between these two results by different measurement methods requires further study.

A recent study from Sadahiro et al. showed that various major surgeries cause changes in DNAm at sites annotated to immune system genes [21]. In this current study, we also demonstrated that neurosurgery alters DNAm levels at many CpG sites on inflammation-related genes. When focusing on the *TNF* gene, levels of DNAm at CpG sites located on the promoter region decreased after neurosurgery [16, 22]. By contrast, levels of DNAm at CpG sites in other regions increased after neurosurgery. In the *IL1B* and the *IL6* gene, levels of DNAm decreased in several CpG sites after neurosurgery. Several studies reported that inflammatory cytokines, including TNF-alpha, IL-1beta, and IL-6, increase after surgery [23, 24]. It is possible that changes of DNAm level after surgery observed in our data are the initial molecular mechanism that causes such elevation of inflammatory cytokines, leading to neuroinflammation, and thus increased risk of delirium [10, 25].

When focusing on each CpG site, there was no significant difference between the POD group and the non-POD group in terms of preoperative DNAm level, postoperative DNAm level, or degree of change by neurosurgery. This might be partly due to the limited sample size. However, in the POD group, we observed at multiple CpG sites that the DNAm level, which varied before surgery, accumulated to a certain narrow range after surgery (Supplementary Fig. 1). Furthermore, it was suggested that scoring of these multiple CpG sites by DNAm level of postoperative blood (IMI) may be useful for the diagnosis of POD (Fig. 1A). The detection ability by IMI was well validated by our independent data set (Fig. 1B). On the other hand, pre-IMI score and diff-IMI score, which were based on DNAm level of preoperative blood and amount of change in DNAm level after surgery, also showed high AUCs, although these methods were not validated as we did not have samples from independent cohorts to validate. Although there are numberless studies investigating delirium biomarkers, those results do not support the use of any one biomarker [26]. This might be because protein level and RNA expression can be easily altered depending on the timing and method of sample collection, storage, and downstream multiple-step laboratory techniques. On the other hand, it is known that DNAm is relatively stable even among various epigenetics mechanisms. Thus, it might be suggested that DNAm can be a potential detection tool for delirium.

Although we analyzed the level of DNAm in peripheral blood samples, it is not clear whether similar changes occur in the brain. In our previous study investigating the relationship between DNAm levels measured at peripheral tissues and brain tissue resected from epilepsy patients, relatively high levels of overall genome-wide DNAm correlation between brain and blood were observed [18]. However, when focusing specifically on the *TNF*, *IL1B*, and *IL6* genes, correlations between brain tissue and blood were not strong. This discrepancy might be due to the fact that the whole brain tissue instead of sorted cells were used to develop the correlation database, “IMAGE-CpG”, in the study [18]. In our other study, all CpGs in the *TNF* gene showed negative correlations between DNAm level and age in glia, but not in neurons [13]. These evidences suggest that it is possible that if we assess the DNAm of each cell type in the brain, especially glial cell, separately, we can better understand the epigenetic status involved in delirium and neuroinflammation, and its relationships to that observed in the blood.

A strength of this work is that it is the first prospective-designed study investigating DNAm from subjects who developed POD from one type of surgery. This is an advantage because potential confounders from different baseline illness types and diverse surgical incisions were avoided. Furthermore, the detection performance of the IMI was validated with two independent cohorts.

We acknowledge several limitations of this study. First, our definition of POD solely depended on retrospective chart reviews of EMRs. Thus, there are certain possibilities of false-positive and false-negative cases in our dataset. However, even with the potential false classification of cases, we observed intriguing signals as outlined above. Second, we have not examined protein levels or mRNA levels of these cytokines, thus the DNAm level discussed above and the subsequent change in cytokine levels and inflammation is speculation, although based on well-accepted molecular processes, and clinical phenotype of POD was associated with this epigenetics data. Third, participants in this study were limited to epilepsy subjects undergoing neurosurgery and the sample size was very small. This was because the number of patients undergoing surgery who could participate in the study was limited. Further study is needed to confirm the present findings using another cohort. Fourth, this study was conducted at a single institution, and greater than 95% of study subjects were non-Hispanic white. Thus, generalizability requires confirmation with a more diverse ethnic population. However, even with these limitations, the present data support evidence of a potential epigenetic contribution to the pathophysiology of POD in the pro-inflammatory cytokine gene.

In summary, this is the first study demonstrating the role of DNAm change among POD patients using both pre- and post-surgery samples. Our findings provide further evidence of the potential role of epigenetics and inflammation in the pathophysiology of delirium.

## Supplementary information

supplementary materials

## Data Availability

The data that support the findings of this study are available from the corresponding author, G.S., upon reasonable request.
