# Supplementary material for: DNA methylation in the inflammatory genes after neurosurgery and diagnostic ability of post-operative delirium
Source: Transl Psychiatry. 2021 Dec 9;11:627. doi: 10.1038/s41398-021-01752-6 (PMC8660911; doi:10.1038/s41398-021-01752-6)
Supplement: Supplementary file 1 — supplementary materials [file 41398_2021_1752_MOESM1_ESM.pdf]

Supplementary method

Calculation of Inflammatory methylation Index (IMI)

Score named “Inflammatory Methylation Index (IMI)” was obtained for each subject. First, ROC curves were made and AUCs were calculated based on the DNA methylation levels of each CpG site. We selected several CpG sites with a large AUC as candidates to construct the combined index. Then, we optimized the number and combination of CpG sites to obtain a better ROC curve, and five CpG sites were selected for the IMI. Five CpG sites: one CpG sites from the *TNF* gene (cg08639424), two CpG sites from the *IL1B* gene (cg15836722 and cg23149881), and two CpG sites from the *IL6* gene (cg15703690 and cg17067544) were selected to calculate IMI. We first created an ROC curve for each CpG site to determine the best cutoff value to separate POD from non-POD. For each CpG site, we added 1 point if the post-operative DNAm level met the criteria divided by the cutoff value, 0 points if it did not, and repeated the process for 5 CpGs (**Supplementary Figure 1**). The total score of those points was used as IMI (ranging from 0 to 5). Then, the relationship between IMI and POD was evaluated by a ROC curve. Two examples are shown below. To validate the results, the IMI was calculated using the same CpG sites and the same process with another cohort from our previous study. Then, the relationship between IMI from the previous cohort and delirium was tested.
Furthermore, the same approach was used to calculate the IMI from the pre-operative DNAm (pre-IMI) and amount of change in DNAm level after surgery (diff-IMI). Five different CpG sites were selected to optimize the ROC; cg23384708 (*TNF* gene), cg07250315 (*IL1B* gene), cg18635064 (*IL1B* gene), cg03601896 (*IL6* gene), and cg10140158 (*IL6* gene) for pre-IMI and cg19124225 (*TNF* gene), cg07250315 (*IL1B* gene), cg20157753 (*IL1B* gene), cg07998387 (*IL6* gene), and cg13104385 (*IL6* gene) for diff-IMI.

Supplementary Figure 1

***TNF* gene: cg08639424**

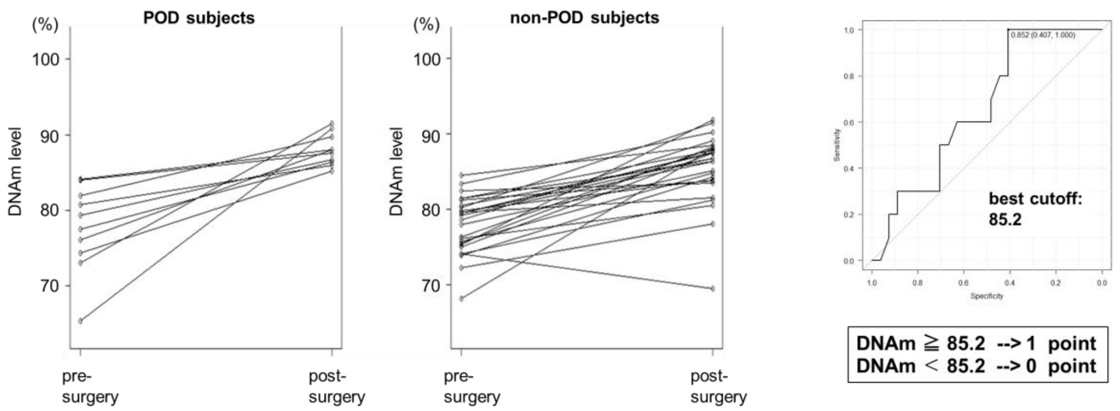

***IL1B* gene: cg15836722**

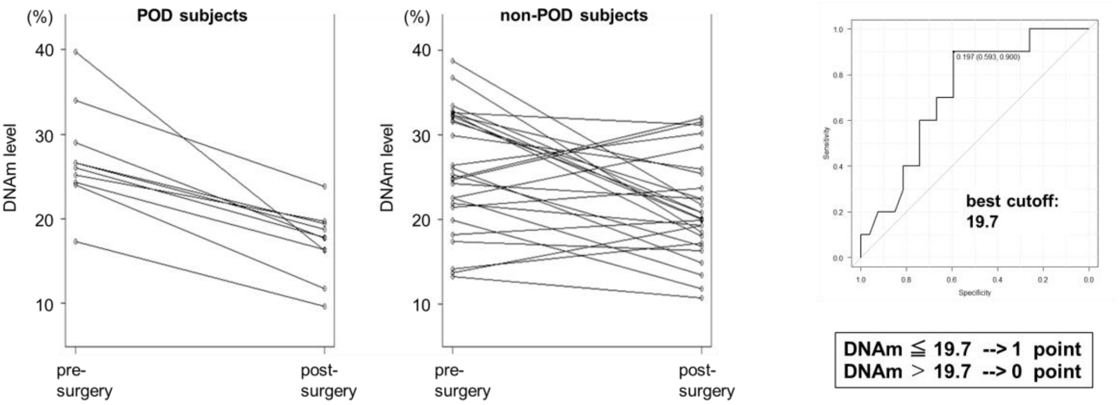

***IL1B* gene: cg23149881**

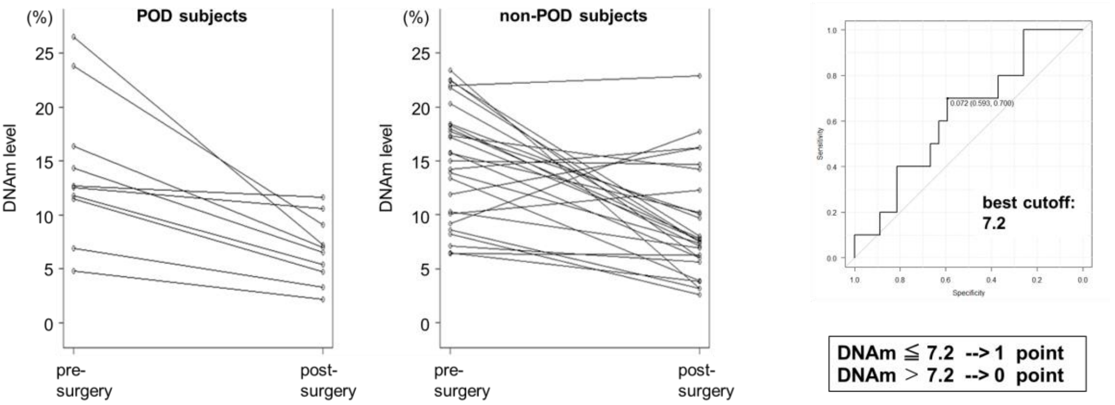

***IL6* gene: cg15703690**

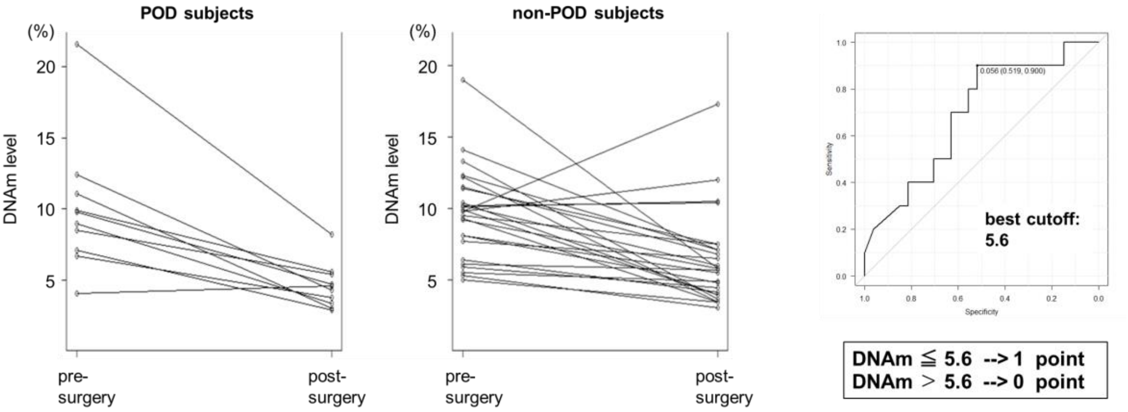

***IL6* gene: cg17067544**

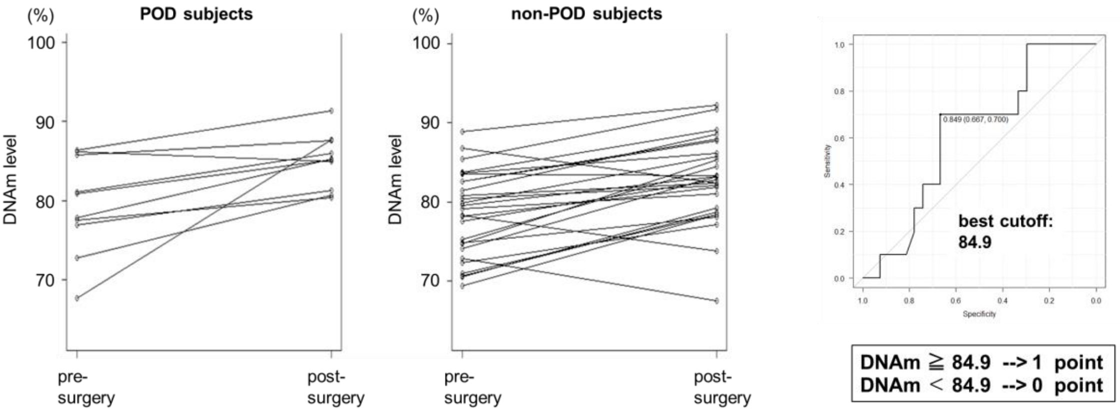

< examples >

		subject A		subject B		
		DNAm (%)	point	DNAm (%)	point	criteria (1 point)
TNF	cg08639424	88.3	1	80.5	0	DNAm >= 85.2
IL1B	cg15836722	13.4	1	31.2	0	DNAm <= 19.7
IL1B	cg23149881	2.6	1	22.9	0	DNAm <= 7.2
IL6	cg15703690	3.4	1	6.8	0	DNAm <= 5.6
IL6	cg17067544	82.4	0	85.7	1	DNAm >= 84.9
	IMI		***4***		***1***	

Supplementary Tables

Supplementary Table 1. Patient Characteristics

Classification	All Subjects	EPIC analysis				pyrosequencing			
		POD	non-POD			POD	non-POD		
N	39	10	27	p	Statistical test	7	20	p	Statistical test
Mean age — yr	33.4	41.0	29.8	0.046	t = -2.07	44.9	29.5	0.01	t = -2.71
SD	15.1	15.6	14.3			15.1	2.1		
Female sex (n)	15	5	8	0.44	χ2 = 0.59	4	7	0.56	χ2 = 0.34
%	38.5	50.0	29.6			57.1	35.0		
Race, White (n)	36	10	26	>0.99	χ2 < 0.01	7	19	>0.99	χ2 < 0.01
%	97.3	100.0	96.3			100.0	95.0		
alcohol (n)	12	3	9	>0.99	χ2 < 0.01	3	8	>0.99	χ2 < 0.01
%	30.8	30.0	33.3			42.9	40.0		
tobacco (n)	16	5	10	0.74	χ2 = 0.11	4	10	>0.99	χ2 < 0.01
%	41.0	50.0	37.0			57.1	50.0		
Mean ASA-PS	2.4	2.2	2.5	0.13	t = 1.56	2.1	2.5	0.16	t = 1.45
SD	0.5	0.4	0.5			0.4	0.5		
Mean BMI	29.8	29.7	29.5	0.96	t = -0.06	32.2	27.2	0.18	t = -1.38
SD	10.4	8.6	11.1			8.6	8.3		
Mean anesthesia time (min)	530.6	533.8	536.7	0.93	t = 0.09	535.1	525.0	0.82	t = -0.23
SD	91.0	88.4	90.0			85.5	104.0		
mean procedure time (min)	348.1	355.3	353.0	0.95	t = -0.07	343.1	339.5	0.93	t = -0.09
SD	90.1	95.4	87.0			81.1	96.2		
Mean blood loss (ml)	165.0	145.0	178.3	0.46	t = 0.74	160.7	186.5	0.69	t = 0.41
SD	120.0	165.3	102.1			199.9	121.2		

Abbreviation: POD; post operative delirium, SD; Standard deviation,
ASA: the American Society of Anesthesiologists (ASA) physical status, BMI; Body mass index

Supplementary Table 2. Cell type proportions

cell type	pre-surgery				post-surgery				amount of change (post-pre)			
	POD	non-POD			POD	non-POD			POD	non-POD		
N	10	27	p	Statistical test	10	27	p	Statistical test	10	27	p	Statistical test
CD8 T cells (%)	6.8	8.6	0.27	t = 1.12	1.6	3.3	0.26	t = 1.16	-5.2	-5.3	0.95	t = -0.06
SD	3.1	4.6			1.6	4.4			3.4	4.5		
CD4-T cells (%)	12.3	11.7	0.79	t = -0.27	0.9	4.0	0.13	t = 1.55	-11.4	-7.7	0.21	t = 1.28
SD	4.6	6.4			0.8	6.2			4.7	8.7		
natural killer cells (%)	0.9	1.3	0.57	t = 0.57	1.1	1.5	0.64	t = 0.47	0.2	0.2	0.97	t = -0.04
SD	1.5	2.1			1.9	2.2			2.5	3.0		
B cells (%)	4.0	2.9	0.31	t = -1.03	0.9	1.6	0.30	t = 1.05	-3.2	-1.4	0.11	t = 1.65
SD	3.3	2.7			1.1	2.0			3.2	2.8		
Monocytes (%)	7.3	7.5	0.84	t = 0.20	3.0	3.6	0.50	t = 0.68	-4.3	-3.9	0.70	t = 0.39
SD	3.2	2.9			1.7	2.6			3.0	2.7		

Abbreviation: POD; post operative delirium, SD; Standard deviation

Supplementary Table 3. Pre-surgery DNAm in the *TNF* gene

		EPIC					pyrosequencing				
		POD	non-POD				POD	non-POD			
		N=10	N=27		t-test	adjusted	N=7	N=20		t-test	adjusted
cg	location	mean	mean	diff	p-value	p-value	mean	mean	diff	p-value	p-value
	chr6: 31542460						72.7	74.5	-1.7	0.30	0.98
cg08639424	chr6: 31542556	77.7	78.0	0.3	0.85	0.90					
cg19978379	chr6: 31542671	73.0	71.3	-1.7	0.37	0.24					
cg24452282	chr6: 31542741	73.8	73.3	-0.5	0.83	0.79					
	chr6: 31543170						6.5	7.2	-0.7	0.59	0.49
	chr6: 31543176						8.0	9.2	-1.2	0.44	0.70
	chr6: 31543178						2.0	3.8	-1.8	0.08	0.56
	chr6: 31543193						4.6	5.5	-0.9	0.47	0.53
cg21370522	chr6: 31543219	13.6	13.0	-0.6	0.75	0.73	3.8	4.4	-0.5	0.50	0.53
cg19648923	chr6: 31543266	10.3	10.6	0.3	0.84	0.47	2.7	3.0	-0.4	0.56	0.71
cg01569083	chr6: 31543290	19.0	18.8	-0.2	0.94	0.47	2.9	3.5	-0.6	0.38	0.62
cg03037030	chr6: 31543300	5.6	6.6	1.0	0.38	0.92	8.5	9.1	-0.7	0.61	0.61
	chr6: 31543430						1.5	2.1	-0.6	0.15	0.70
	chr6: 31543487						5.3	6.6	-1.3	0.20	0.81
cg12681001	chr6: 31543541	8.8	9.1	0.4	0.79	0.39	9.1	10.9	-1.8	0.20	0.67
cg21222743	chr6: 31543546	5.8	6.9	1.1	0.39	0.49	7.7	9.2	-1.5	0.22	0.80
cg10717214	chr6: 31543558	8.3	9.7	1.4	0.33	0.46	33.6	33.5	0.1	0.96	0.83
cg04425624	chr6: 31543566	17.2	17.5	0.3	0.92	0.30	20.8	21.3	-0.5	0.86	0.44
cg21467614	chr6: 31543638	17.1	17.9	0.8	0.66	0.18					
cg08553327	chr6: 31543647	15.4	16.9	1.6	0.53	0.31					
cg26729380	chr6: 31543655	10.6	11.9	1.3	0.49	0.14					
cg10650821	chr6: 31543686	11.4	11.7	0.3	0.86	0.58					
	chr6: 31544695						63.2	66.1	-2.9	0.59	0.60
	chr6: 31544752						78.2	76.8	1.4	0.70	0.46
	chr6: 31544800						24.0	22.3	1.8	0.26	0.17
	chr6: 31544822						58.1	56.0	2.1	0.40	0.30
	chr6: 31544848						14.6	12.8	1.7	0.09	0.01
cg01360627	chr6: 31544931	69.8	70.0	0.2	0.93	0.97	27.4	25.3	2.1	0.23	0.10
cg23384708	chr6: 31544934	67.4	63.0	-4.4	0.04	0.77	77.3	72.8	4.5	0.16	0.11
cg20477259	chr6: 31544960	62.5	61.3	-1.2	0.61	0.78	68.9	67.5	1.4	0.63	0.65
	chr6: 31545253						87.9	88.2	-0.3	0.88	0.91
	chr6: 31545258						88.7	87.4	1.3	0.52	0.31
	chr6: 31545267						84.1	81.3	2.8	0.29	0.14
cg15989608	chr6: 31545322	72.2	70.9	-1.3	0.54	0.35	92.1	89.8	2.3	0.21	0.12
cg26736341	chr6: 31545343	71.4	69.5	-1.8	0.43	0.35	78.0	79.2	-1.1	0.65	0.21
	chr6: 31545432						89.9	85.9	3.9	0.11	0.09
cg04472685	chr6: 31545474	82.0	81.2	-0.7	0.66	0.56	83.4	84.2	-0.8	0.68	0.77
cg19124225	chr6: 31545836	94.8	95.9	1.1	0.10	0.45					
cg02137984	chr6: 31545899	93.7	95.1	1.4	0.10	0.27					
cg06825478	chr6: 31546068	91.0	89.9	-1.2	0.47	0.28	91.8	91.8	0.1	0.93	0.64
	chr6: 31546086						85.2	85.1	0.1	0.96	0.97

Notes:
underline nominal significance ($P < 0.05$)
adjusted p-value (EPIC analysis); multiple regression analysis results corrected by age, sex, and cell proportion.
adjusted p-value (pyrosequencing); multiple regression analysis results corrected by age and sex.

Supplementary Table 4. Pre-surgery DNAm in the *IL1B* and *IL6* gene

cg	POD N=10 mean	non-POD N=27 mean	diff	t-test p-value	adjusted p-value
IL1B					
cg01290568	26.2	26.5	0.3	0.92	0.59
cg02596281	84.4	84.2	-0.2	0.93	0.78
cg07250315	39.9	36.9	-3.0	0.16	0.02
cg07935264	8.8	9.2	0.4	0.70	0.78
cg10486274	93.9	93.6	-0.3	0.65	0.64
cg14117934	86.7	86.1	-0.6	0.73	0.30
cg15218327	92.4	92.0	-0.4	0.69	0.36
cg15836722	27.3	26.0	-1.3	0.61	0.28
cg18635064	93.2	95.1	1.9	0.14	0.10
cg18773937	6.8	7.3	0.6	0.65	0.57
cg19890119	15.9	15.5	-0.5	0.83	0.97
cg20157753	5.9	5.2	-0.7	0.19	0.17
cg20983042	87.5	86.9	-0.6	0.77	0.85
cg23149881	14.1	15.0	0.9	0.67	0.52
IL6					
cg00087425	6.3	6.5	0.2	0.72	0.96
cg00175482	88.6	89.1	0.6	0.62	0.78
cg01770232	7.7	6.9	-0.8	0.44	0.98
cg02335517	93.3	93.4	0.2	0.87	0.46
cg03601896	4.0	3.7	-0.4	0.57	> 0.99
cg05265849	21.4	19.7	-1.7	0.56	0.62
cg07998387	69.8	69.4	-0.5	0.80	0.79
cg10140158	86.8	88.0	1.2	0.34	0.37
cg13104385	41.1	39.5	-1.6	0.67	0.66
cg15703690	10.0	9.5	-0.5	0.69	0.42
cg17067544	79.3	78.5	-0.8	0.69	0.53
cg20509117	62.2	62.3	0.1	0.98	0.83
cg21785978	5.2	5.5	0.3	0.68	0.84
cg23731304	38.7	37.2	-1.6	0.48	0.11

Notes:
adjusted p-value; multiple regression analysis results corrected by age, sex, and cell proportion.

Supplementary Table 5. Correlations between age and pre-surgery DNAm in the *IL1B* and *IL6* gene

	all N=37		POD N=10		non-POD N=27	
cg	r	p	r	p	r	p
IL1B						
cg01290568	-0.16	0.35	-0.08	0.84	-0.19	0.35
cg02596281	-0.11	0.53	-0.12	0.74	-0.12	0.56
cg07250315	-0.02	0.91	0.05	0.89	-0.16	0.43
cg07935264	-0.25	0.14	-0.33	0.35	-0.19	0.34
cg10486274	-0.05	0.75	-0.29	0.42	0.01	0.96
cg14117934	-0.22	0.20	0.48	0.16	-0.40	0.04
cg15218327	-0.27	0.10	-0.75	0.01	-0.21	0.30
cg15836722	-0.14	0.42	-0.16	0.65	-0.18	0.36
cg18635064	-0.11	0.53	-0.17	0.65	0.05	0.79
cg18773937	-0.22	0.20	0.34	0.34	-0.42	0.03
cg19890119	0.06	0.73	0.14	0.71	0.02	0.93
cg20157753	0.00	0.98	-0.40	0.26	0.04	0.86
cg20983042	-0.13	0.45	-0.17	0.64	-0.15	0.46
cg23149881	-0.25	0.13	-0.22	0.53	-0.25	0.21
IL6						
cg00087425	-0.02	0.90	-0.18	0.62	0.05	0.81
cg00175482	-0.24	0.16	-0.33	0.35	-0.16	0.42
cg01770232	0.14	0.39	0.48	0.16	-0.17	0.41
cg02335517	0.08	0.64	0.50	0.14	0.01	0.95
cg03601896	0.29	0.08	0.49	0.15	0.18	0.37
cg05265849	0.14	0.39	0.16	0.65	0.10	0.63
cg07998387	-0.06	0.73	0.24	0.50	-0.18	0.38
cg10140158	-0.20	0.25	-0.26	0.46	-0.11	0.59
cg13104385	0.04	0.84	0.13	0.73	-0.04	0.85
cg15703690	0.38	0.02	0.55	0.10	0.29	0.14
cg17067544	-0.24	0.15	-0.24	0.50	-0.30	0.14
cg20509117	0.14	0.41	0.47	0.17	0.07	0.75
cg21785978	-0.38	0.02	-0.11	0.77	-0.49	0.01
cg23731304	-0.33	0.04	-0.32	0.37	-0.43	0.03

Notes:
underline nominal significance (P < 0.05)

Supplementary Table 6. Post-surgery DNAm in the *TNF* gene

		EPIC					pyrosequencing				
		POD	non-POD				POD	non-POD			
		N=10	N=27		t-test	adjusted	N=7	N=20		t-test	adjusted
cg	location	mean	mean	diff	p-value	p-value	mean	mean	diff	p-value	p-value
	chr6: 31542460						78.1	78.1	0.0	0.99	0.84
cg08639424	chr6: 31542556	88.0	85.5	2.5	0.11	0.28					
cg19978379	chr6: 31542671	81.9	80.6	1.3	0.55	0.11					
cg24452282	chr6: 31542741	86.9	83.6	3.3	0.23	0.53					
	chr6: 31543170						6.4	5.4	1.0	0.30	0.03
	chr6: 31543176						7.5	7.6	-0.1	0.90	0.27
	chr6: 31543178						2.3	2.7	-0.4	0.50	0.84
	chr6: 31543193						3.5	4.1	-0.6	0.45	0.62
cg21370522	chr6: 31543219	14.4	13.9	0.5	0.84	0.60	3.6	3.4	0.2	0.63	0.11
cg19648923	chr6: 31543266	7.8	8.2	-0.4	0.76	0.59	2.4	2.3	0.1	0.82	0.32
cg01569083	chr6: 31543290	16.8	17.7	-0.9	0.65	0.98	2.5	2.7	-0.2	0.61	0.46
cg03037030	chr6: 31543300	4.5	5.0	-0.5	0.59	0.77	7.6	7.8	-0.3	0.74	0.65
	chr6: 31543430						1.9	2.0	-0.1	0.80	0.34
	chr6: 31543487						4.9	5.4	-0.5	0.64	0.61
cg12681001	chr6: 31543541	6.7	7.5	-0.7	0.30	0.93	9.5	9.0	0.5	0.75	0.50
cg21222743	chr6: 31543546	3.4	4.2	-0.9	0.26	0.01	7.1	7.4	-0.3	0.74	0.60
cg10717214	chr6: 31543558	6.0	6.5	-0.5	0.62	0.02	35.0	32.0	3.1	0.35	0.90
cg04425624	chr6: 31543566	12.0	13.5	-1.5	0.42	0.64	21.6	20.5	1.1	0.70	0.86
cg21467614	chr6: 31543638	14.1	15.0	-0.9	0.47	0.23					
cg08553327	chr6: 31543647	11.6	13.7	-2.1	0.24	0.65					
cg26729380	chr6: 31543655	6.4	8.4	-2.1	0.13	0.44					
cg10650821	chr6: 31543686	10.0	10.2	-0.2	0.86	0.63					
	chr6: 31544695						69.4	73.5	-4.0	0.50	0.43
	chr6: 31544752						86.2	85.9	0.3	0.92	0.96
	chr6: 31544800						26.3	23.8	2.5	0.08	0.21
	chr6: 31544822						62.5	62.1	0.4	0.88	0.75
	chr6: 31544848						14.0	13.0	1.0	0.14	0.06
cg01360627	chr6: 31544931	80.2	78.1	2.1	0.37	0.69	28.9	26.7	2.2	0.22	0.34
cg23384708	chr6: 31544934	74.0	71.6	2.4	0.32	0.47	83.9	82.0	1.8	0.56	0.90
cg20477259	chr6: 31544960	73.4	71.3	2.1	0.44	0.42	76.4	74.1	2.4	0.37	0.74
	chr6: 31545253						92.3	91.8	0.5	0.78	0.93
	chr6: 31545258						92.2	91.3	0.9	0.73	0.72
	chr6: 31545267						87.8	87.8	0.0	>0.99	0.68
cg15989608	chr6: 31545322	80.8	79.1	1.7	0.43	0.49	91.9	94.2	-2.3	0.10	0.03
cg26736341	chr6: 31545343	80.6	79.0	1.7	0.48	0.61	82.4	80.4	2.0	0.37	0.39
	chr6: 31545432						91.4	89.1	2.2	0.57	0.66
cg04472685	chr6: 31545474	87.9	87.4	0.5	0.73	0.14	87.1	86.7	0.4	0.82	0.62
cg19124225	chr6: 31545836	96.5	96.1	0.3	0.50	0.98					
cg02137984	chr6: 31545899	96.8	96.5	0.2	0.69	0.28					
cg06825478	chr6: 31546068	94.7	94.1	0.6	0.59	0.77	94.5	94.2	0.3	0.70	0.70
	chr6: 31546086						89.4	89.3	0.1	0.91	0.73

Notes:
underline nominal significance (P < 0.05)
adjusted p-value (EPIC analysis); multiple regression analysis results corrected by age, sex, and cell proportion.
adjusted p-value (pyrosequencing); multiple regression analysis results corrected by age and sex.

Supplementary Table 7. Post-surgery DNAm in the *IL1B* and *IL6* gene

cg	POD N=10 mean	non-POD N=27 mean	diff	t-test p-value	adjusted p-value
IL1B					
cg01290568	12.7	17.3	-4.6	0.09	0.58
cg02596281	81.8	82.9	-1.1	0.47	0.82
cg07250315	33.0	33.3	-0.3	0.88	0.17
cg07935264	5.5	6.8	-1.2	0.20	0.73
cg10486274	93.1	93.1	0.1	0.91	0.71
cg14117934	85.8	85.4	0.3	0.76	0.87
cg15218327	89.6	88.2	1.4	0.18	<u>0.02</u>
cg15836722	17.1	21.2	-4.1	0.05	0.12
cg18635064	92.4	95.0	-2.6	<u>0.04</u>	0.05
cg18773937	4.2	5.1	-0.9	0.27	0.91
cg19890119	6.7	9.6	-2.9	0.16	0.69
cg20157753	4.2	5.0	-0.8	0.13	0.61
cg20983042	86.8	87.1	-0.3	0.80	0.74
cg23149881	6.8	9.1	-2.3	0.19	0.69
IL6					
cg00087425	6.2	6.8	-0.6	0.40	0.58
cg00175482	85.7	87.3	-1.6	0.22	0.48
cg01770232	7.7	7.5	0.2	0.77	0.50
cg02335517	93.0	92.5	0.4	0.63	0.88
cg03601896	2.7	3.1	-0.4	0.41	0.60
cg05265849	16.9	17.9	-1.0	0.72	0.68
cg07998387	68.6	70.7	-2.2	0.29	0.50
cg10140158	87.0	88.2	-1.2	0.20	0.38
cg13104385	38.4	39.4	-0.9	0.81	0.63
cg15703690	4.6	6.2	-1.7	0.13	0.31
cg17067544	85.0	82.7	2.3	0.22	0.59
cg20509117	62.5	61.6	0.8	0.78	0.96
cg21785978	4.4	5.3	-0.9	0.15	0.08
cg23731304	33.5	34.9	-1.4	0.49	0.98

Notes:
<u>underline</u> nominal significance ($P < 0.05$)
adjusted p-value; multiple regression analysis results corrected by age, sex, and cell proportion.

Supplementary Table 8. Correlations between age and post-surgery DNAm in the *IL1B* and *IL6* gene

	all N=37		POD N=10		non-POD N=27	
cg	r	p	r	p	r	p
IL1B						
cg01290568	-0.08	0.65	-0.21	0.57	0.04	0.83
cg02596281	-0.09	0.58	0.06	0.86	-0.11	0.58
cg07250315	-0.09	0.61	-0.03	0.94	-0.10	0.63
cg07935264	-0.06	0.72	-0.53	0.11	0.16	0.44
cg10486274	-0.14	0.42	0.02	0.96	-0.19	0.33
cg14117934	-0.05	0.75	0.67	0.03	-0.33	0.09
cg15218327	-0.11	0.52	0.10	0.79	-0.28	0.15
cg15836722	0.00	0.98	0.11	0.77	0.12	0.55
cg18635064	-0.10	0.55	0.16	0.66	-0.10	0.63
cg18773937	-0.13	0.46	0.06	0.87	-0.11	0.59
cg19890119	0.10	0.56	-0.01	0.98	0.24	0.23
cg20157753	-0.09	0.59	-0.55	0.10	0.22	0.27
cg20983042	-0.07	0.69	0.68	0.03	-0.32	0.10
cg23149881	-0.12	0.46	-0.25	0.50	-0.01	0.95
IL6						
cg00087425	0.34	<u>0.04</u>	0.02	0.95	0.56	<u>0.003</u>
cg00175482	-0.22	0.19	0.03	0.94	-0.32	0.11
cg01770232	0.26	0.12	0.36	0.30	0.22	0.28
cg02335517	0.06	0.71	0.59	0.07	-0.08	0.70
cg03601896	0.28	0.09	0.21	0.55	0.40	0.04
cg05265849	0.09	0.59	0.11	0.75	0.12	0.54
cg07998387	-0.20	0.23	-0.19	0.59	-0.14	0.48
cg10140158	-0.07	0.70	-0.23	0.52	0.08	0.70
cg13104385	0.05	0.77	0.10	0.79	0.05	0.79
cg15703690	0.14	0.39	0.18	0.61	0.28	0.16
cg17067544	-0.23	0.17	-0.23	0.52	-0.36	0.07
cg20509117	0.15	0.38	0.29	0.41	0.06	0.75
cg21785978	0.11	0.51	0.47	0.17	0.13	0.53
cg23731304	-0.25	0.13	0.15	0.68	-0.32	0.10

Notes:
<u>underline</u> nominal significance ($P < 0.05$)

Supplementary Table 9. Comparisons degree of DNAm change by neurosurgery between POD and non-POD in the *TNF* gene

		EPIC					pyrosequencing				
		POD	non-POD				POD	non-POD			
		N=10	N=27				N=7	N=20			
cg	location	post-pre mean	post-pre mean	diff	t-test p-value	adjusted p-value	post-pre mean	post-pre mean	diff	t-test p-value	t-test p-value
	chr6: 31542460						5.4	3.6	1.8	0.45	0.87
cg08639424	chr6: 31542556	10.3	7.5	2.8	0.18	0.57					
cg19978379	chr6: 31542671	8.9	9.3	-0.4	0.86	0.17					
cg24452282	chr6: 31542741	13.1	10.3	2.8	0.31	0.86					
	chr6: 31543170						-0.1	-1.8	1.7	0.19	0.40
	chr6: 31543176						-0.5	-1.6	1.1	0.43	0.81
	chr6: 31543178						0.3	-1.1	1.3	0.23	0.72
	chr6: 31543193						-1.1	-1.3	0.2	0.83	0.80
cg21370522	chr6: 31543219	0.8	1.0	-0.2	0.94	0.58	-0.2	-0.9	0.7	0.29	0.72
cg19648923	chr6: 31543266	-2.5	-2.4	-0.1	0.96	0.69	-0.3	-0.8	0.5	0.42	0.73
cg01569083	chr6: 31543290	-2.2	-1.2	-1.1	0.63	0.48	-0.4	-0.8	0.4	0.56	0.97
cg03037030	chr6: 31543300	-1.1	-1.6	0.5	0.66	0.90	-0.9	-1.3	0.4	0.74	0.80
	chr6: 31543430						0.4	-0.1	0.6	0.26	0.38
	chr6: 31543487						-0.4	-1.2	0.8	0.48	0.52
cg12681001	chr6: 31543541	-2.0	-1.7	-0.4	0.78	0.51	0.4	-1.9	2.3	0.11	0.29
cg21222743	chr6: 31543546	-2.4	-2.7	0.2	0.83	0.47	-0.6	-1.8	1.2	0.36	0.56
cg10717214	chr6: 31543558	-2.2	-3.1	0.9	0.47	0.07	1.1	-1.5	2.6	0.38	0.32
cg04425624	chr6: 31543566	-5.2	-3.9	-1.3	0.61	0.51	1.6	-0.8	2.3	0.46	0.55
cg21467614	chr6: 31543638	-3.0	-2.9	-0.1	0.94	0.50					
cg08553327	chr6: 31543647	-3.8	-3.2	-0.5	0.81	0.85					
cg26729380	chr6: 31543655	-4.2	-3.5	-0.7	0.69	0.69					
cg10650821	chr6: 31543686	-1.4	-1.5	0.1	0.97	0.64					
	chr6: 31544695						6.2	7.4	-1.2	0.84	0.73
	chr6: 31544752						8.0	9.0	-1.1	0.81	0.53
	chr6: 31544800						2.3	1.5	0.8	0.59	0.78
	chr6: 31544822						4.4	6.1	-1.7	0.61	0.28
	chr6: 31544848						-0.6	0.2	-0.7	0.29	0.04
cg01360627	chr6: 31544931	10.3	8.0	2.3	0.36	0.58	1.5	1.4	0.1	0.93	0.47
cg23384708	chr6: 31544934	6.7	8.6	-2.0	0.43	<u>0.01</u>	6.6	9.2	-2.6	0.56	0.22
cg20477259	chr6: 31544960	10.9	10.0	0.9	0.73	0.27	7.5	6.6	0.9	0.82	0.89
	chr6: 31545253						4.4	3.6	0.8	0.77	0.89
	chr6: 31545258						3.5	3.9	-0.4	0.89	0.72
	chr6: 31545267						3.7	6.5	-2.8	0.41	0.16
cg15989608	chr6: 31545322	8.7	8.2	0.4	0.86	0.10	-0.7	4.4	-5.1	0.06	<u>0.02</u>
cg26736341	chr6: 31545343	9.3	9.4	-0.1	0.95	0.09	4.6	2.1	2.6	0.40	0.31
	chr6: 31545432						1.5	3.2	-1.7	0.71	0.65
cg04472685	chr6: 31545474	6.0	6.2	-0.2	0.90	0.12	3.1	3.0	0.2	0.94	0.87
cg19124225	chr6: 31545836	1.7	0.2	1.5	0.11	0.52					
cg02137984	chr6: 31545899	3.1	1.4	1.7	0.09	0.92					
cg06825478	chr6: 31546068	3.6	4.2	-0.6	0.77	0.17	2.6	2.4	0.2	0.84	0.96
	chr6: 31546086						4.3	4.2	0.1	0.97	0.81

Notes:
underline nominal significance (P < 0.05)
adjusted p-value (EPIC analysis); multiple regression analysis results corrected by age, sex, and cell proportion.
adjusted p-value (pyrosequencing); multiple regression analysis results corrected by age and sex.

Supplementary Table 10. Comparisons of degree of DNAm change by neurosurgery between POD and non-POD in the IL1B and IL6 gene

cg	POD N=10 post-pre mean	non-POD N=27 post-pre mean	diff	t-test p-value	adjusted p-value
IL1B					
cg01290568	-13.4	-9.1	-4.3	0.23	0.90
cg02596281	-2.6	-1.3	-1.2	0.41	0.90
cg07250315	-6.9	-3.6	-3.3	0.11	0.59
cg07935264	-3.2	-2.4	-0.9	0.52	0.36
cg10486274	-0.7	-0.5	-0.2	0.77	0.63
cg14117934	-0.9	-0.7	-0.2	0.91	0.64
cg15218327	-2.8	-3.7	1.0	0.45	0.66
cg15836722	-10.1	-4.7	-5.4	<u>0.046</u>	<u>0.04</u>
cg18635064	-0.8	-0.1	-0.7	0.36	0.63
cg18773937	-2.5	-2.3	-0.3	0.82	0.25
cg19890119	-9.2	-5.9	-3.4	0.19	0.96
cg20157753	-1.7	-0.2	-1.5	<u>0.03</u>	0.25
cg20983042	-0.6	0.2	-0.8	0.60	0.98
cg23149881	-7.4	-6.0	-1.4	0.57	0.27
IL6					
cg00087425	-0.1	0.3	-0.4	0.64	0.28
cg00175482	-2.9	-1.9	-1.0	0.50	0.66
cg01770232	0.1	0.6	-0.6	0.37	0.67
cg02335517	-0.3	-0.9	0.6	0.35	0.29
cg03601896	-1.3	-0.6	-0.8	0.28	0.76
cg05265849	-4.5	-1.8	-2.7	<u>0.04</u>	0.28
cg07998387	-1.3	1.4	-2.7	<u>0.04</u>	0.31
cg10140158	0.2	0.2	0.0	>0.99	0.93
cg13104385	-2.6	-0.1	-2.5	<u>0.04</u>	0.13
cg15703690	-5.4	-3.2	-2.2	0.15	0.85
cg17067544	5.7	4.2	1.5	0.39	0.75
cg20509117	0.2	-0.7	0.9	0.70	0.40
cg21785978	-0.8	-0.1	-0.6	0.45	0.18
cg23731304	-5.2	-2.3	-3.0	0.08	0.23

Notes:
underline nominal significance (P < 0.05)
adjusted p-value; multiple regression analysis results corrected by age, sex, and cell proportion.

Supplementary Figures

Supplementary Figure 1 is shown in "Supplementary method" section.

Supplementary Figure 2

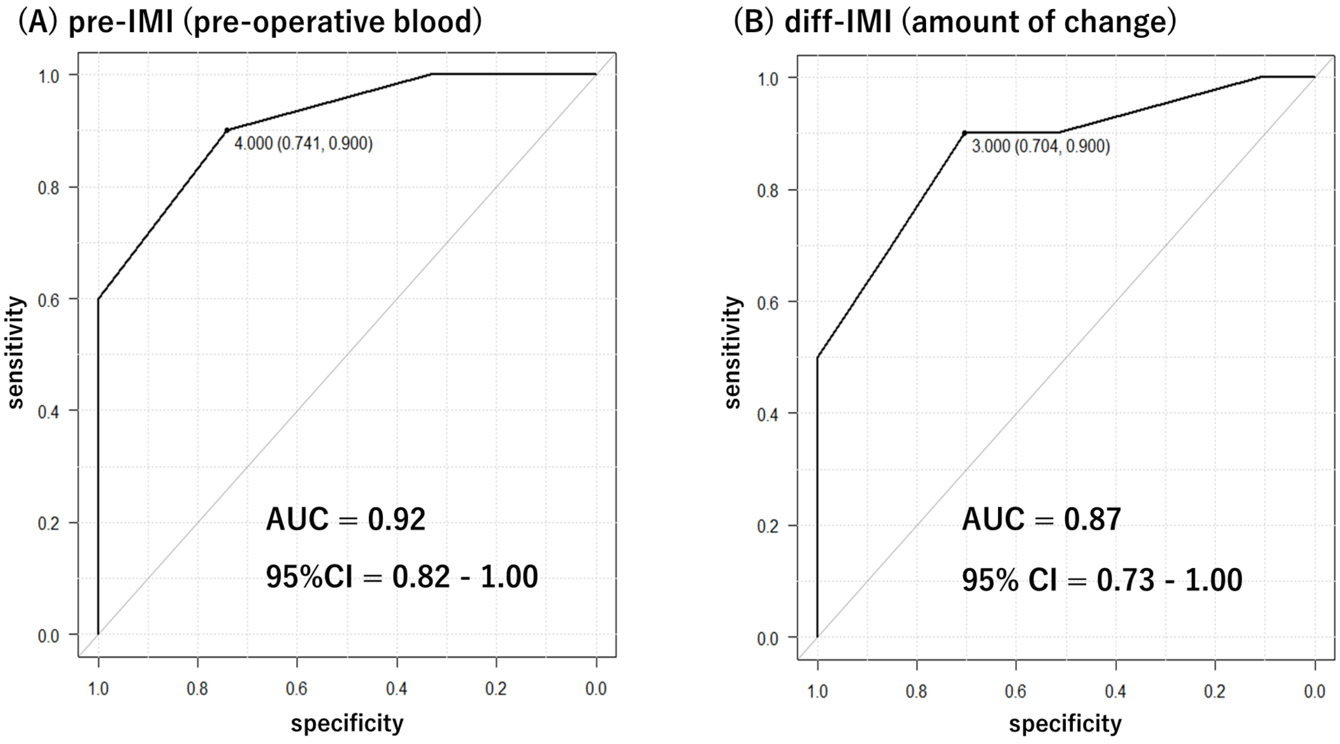

Supplementary Figure 2

(A) ROC curve based on pre-IMI from pre-operative blood

(B) ROC curve based on diff-IMI from amount of change of DNAm

Supplementary Figure 3

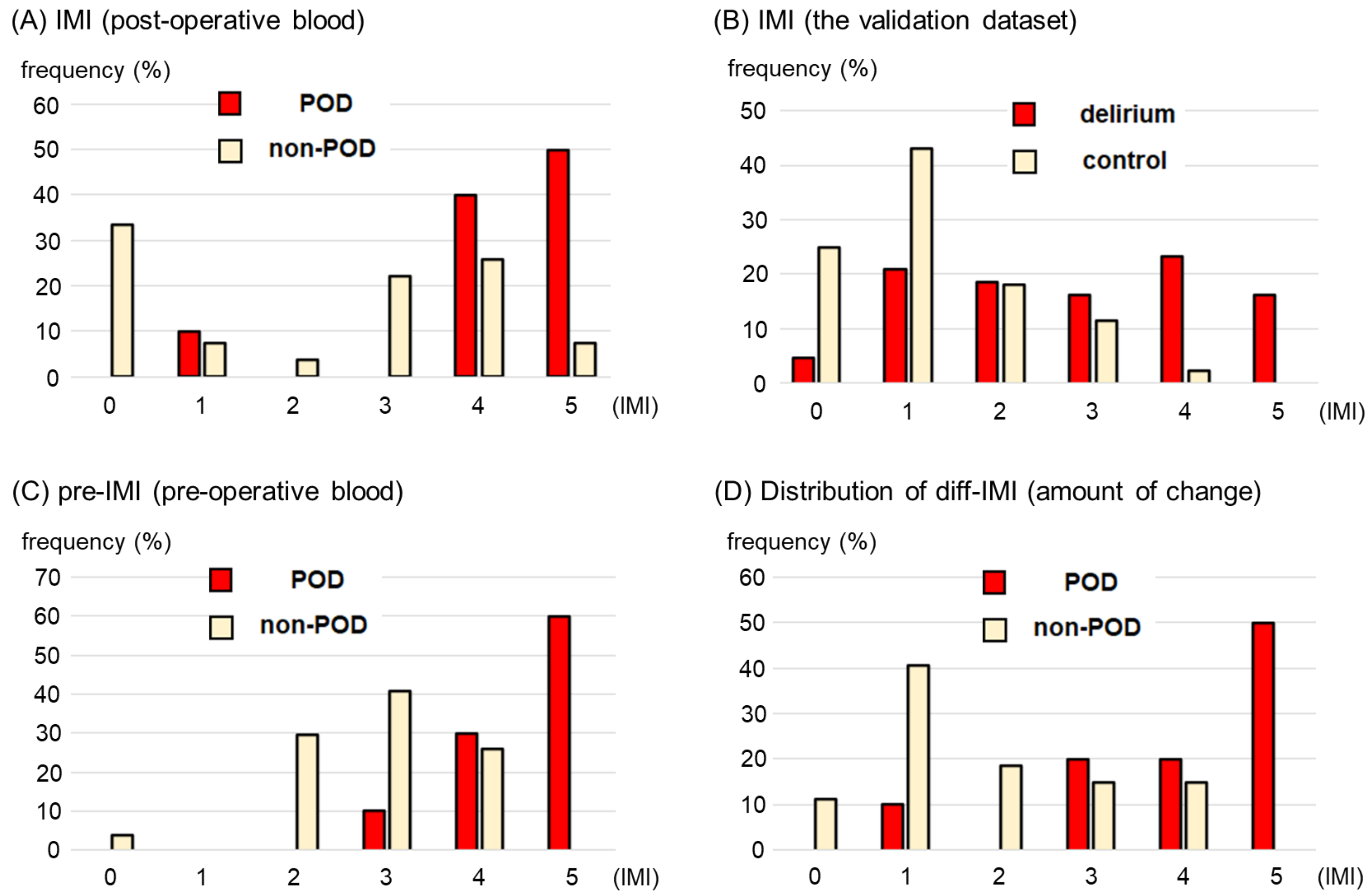

Supplementary Figure 3

(A) Distribution of IMI (post-operative blood)

(B) Distribution of IMI (the validation dataset)

(C) Distribution of pre-IMI (pre-operative blood)

(D) Distribution of diff-IMI (amount of change)
